# Slowed gastrointestinal transit is associated with an altered caecal microbiota in an aged rat model

**DOI:** 10.3389/fcimb.2023.1139152

**Published:** 2023-03-14

**Authors:** Nabil Parkar, Julie E. Dalziel, Nick J. Spencer, Patrick Janssen, Warren C. McNabb, Wayne Young

**Affiliations:** ^1^ Smart Foods and Bioproducts, AgResearch Smart, Palmerston North, New Zealand; ^2^ Riddet Institute, Massey University, Palmerston North, New Zealand; ^3^ School of Food and Advanced Technology, Massey University, Palmerston North, New Zealand; ^4^ Discipline of Physiology, College of Medicine and Public Health, Flinders University, School of Medicine, Adelaide, SA, Australia

**Keywords:** intestinal motility, enteric nervous system, loperamide, Bacteroides, caecal microbiota

## Abstract

Gastrointestinal (GI) motility is largely dependent upon activity within the enteric nervous system (ENS) and is an important part of the digestive process. Dysfunction of the ENS can impair GI motility as is seen in the case of constipation where gut transit time is prolonged. Animal models mimicking symptoms of constipation have been developed by way of pharmacological manipulations. Studies have reported an association between altered GI motility and gut microbial population. Little is known about the changes in gut microbiota profile resulting specifically from pharmacologically induced slowed GI motility in rats. Moreover, the relationship between gut microbiota and altered intestinal motility is based on studies using faecal samples, which are easier to obtain but do not accurately reflect the intestinal microbiome. The aim of this study was to examine how delayed GI transit due to opioid receptor agonism in the ENS modifies caecal microbiota composition. Differences in caecal microbial composition of loperamide-treated or control male Sprague Dawley rats were determined by 16S rRNA gene amplicon sequencing. The results revealed that significant differences were observed at both genus and family level between treatment groups. *Bacteroides* were relatively abundant in the loperamide-induced slowed GI transit group, compared to controls. Richness and diversity of the bacterial communities was significantly lower in the loperamide-treated group compared to the control group. Understanding the link between specific microbial species and varying transit times is crucial to design interventions targeting the microbiome and to treat intestinal motility disorders.

## 1 Introduction

Gastrointestinal (GI) motility is an integral part of digestive function. The enteric nervous system (ENS) plays a major role in control of GI motility (Spencer and Hu, 2020). Gut transit time, which refers to the transit of luminal content along the GI tract, is commonly used as a marker of gut motility and function (Corsetti et al., 2019). Measurement of gut transit time is relevant when addressing GI motility disorders such as irritable bowel syndrome (IBS) and constipation.

The mammalian GI tract is colonized by a diverse population of microbial communities. These gut microbes are vital in maintaining host health (Thursby and Juge, 2017). Numerous studies have pointed out that an association exists between the gut microbiota and GI motility, and that this relationship is likely to be bidirectional (Quigley, 2011; Zhang et al., 2021). Experiments using germ free (GF) animal models have demonstrated that a lack of microbial colonization correlates with altered ENS functions such as delayed gastric emptying and slowed GI transit (Hyland and Cryan, 2016). Conversely, alterations in GI motility can modify the resident microbial population as seen in the case of Small Intestinal Bacterial Overgrowth (SIBO), a clinical syndrome often associated with altered GI motility (Toskes, 1993). Environmental factors have also been shown to influence gut microbiota composition. In this context, it can be considered that changes in gut motility likely led to changes in microbiota composition and function. For example, some microbial taxa benefit from increases in GI motility, relative to other species adapted to conditions associated with slower motility (Pianka, 1970). This concept is consistent with ecological principles of r/K selection in response to environmental disturbance (Pianka, 1970). As GI transit time decreases, such as with diarrhea, species better adapted to grow rapidly during reduced competition (r-selected) will dominate the gut. In contrast, prolonged colonic transit may facilitate the amplification and colonization of slow-growing species, better adapted to persist in competitive environments (K-selected); these species include metabolically economical taxa. The direct effects of gut motility on specific microbial communities could cascade into broad ecosystem changes as the community is interconnected metabolically. The question of identifying which microbial species are impacted by motility is important because shifts from normal microbiota composition can lead to metabolic changes with possible physiological consequences.

Animal models mimicking symptoms of constipation have been developed by way of pharmacological manipulations. Loperamide is an opioid receptor agonist that works by activating the µ-opioid receptors located in the myenteric plexus of the ENS (Kim et al., 2014). It does not cross the blood brain barrier (Montesinos et al., 2014). Upon binding to the opioid receptors, loperamide decreases the activity of the myenteric plexus, which subsequently reduces the tone of the circular and longitudinal smooth muscles of the gut wall. This in turn reduces propulsion and extends the total stay time of luminal contents (Kim et al., 2014). Through our experiments we have previously shown that loperamide works by inhibiting enteric neuronal activity, delays GI transit, inducing constipation in aged rats (Dalziel et al., 2016). Similarly other studies have reported that loperamide inhibits colonic peristalsis and intestinal water secretion causing delayed GI transit time (Hughes et al., 1984; Wintola et al., 2010). Loperamide-induced slowed transit is therefore considered to be a model of spastic constipation due to increased colonic contractions and inhibition of stool frequency (Takasaki et al., 1994). Although the dose and time for loperamide administration has been described to vary among rodent studies, loperamide has shown to be effective in inducing constipation when administered subcutaneously (Chen et al., 2012; Lee et al., 2012; Neri et al., 2012), orally (Wintola et al., 2010) or intra-peritoneally (Jeon and Choi, 2010) at doses ranging from 0.2 to 5 mg/kg body weight for 3 to 7 days. Although longer transit times have been associated with taxa such as *Akkermansia*, *Bacteroides* and *Alistipes* (Asnicar et al., 2021), little is known about the changes in gut microbiota profile resulting specifically from pharmacologically-induced slowed GI motility in rats. Moreover, the relationship between gut microbiota and altered intestinal motility are based on studies using faecal samples, which are easier to obtain but do not accurately reflect the intestinal microbial community (Wang et al., 2019). The caecum serves as a bacterial reservoir that populates the large intestine (Brown et al., 2018). The aim of this study was to examine how slowed GI transit due to opioid receptor agonism in the ENS affects the caecal microbiota.

## 2 Materials and methods

### 2.1 Animals

Male Sprague Dawley rats were bred at the AgResearch Ruakura Small Animal Unit (Hamilton, New Zealand) and raised in group housing with littermates to 18 months of age (804 ± 13 g) (Dalziel et al., 2017). The rats were maintained under a 12-h light/dark cycle with water and food provided ad libitum. Rats were fed a nutritionally balanced diet (OpenStandard Rodent Diet, Research Diets, Inc., New Brunswick, NJ, USA) as previously described (Dalziel et al., 2017). They were monitored three times weekly for General Health Score (1–5; NZ Animal Health Care Standard), weight and food intake. The experiment was performed in accordance with the Animal Welfare Act, 1999 (NZ). The protocol was approved by the AgResearch Grasslands Animal Ethics Committee (Ethics approval No.: AE12933).

### 2.2 Study design and pharmacological treatment

This study included a loperamide-treated group and a control group with age and weight balanced amongst treatment groups. Loperamide hydrochloride (S2480) was purchased from Selleck Chemicals (Houston, TX, USA). Rats were administered 1 mg/kg/day loperamide (in 100% Dimethyl sulfoxide (DMSO) or DMSO Vehicle only (control) for seven days. The drug dose has been previously determined to be effective over seven days (Dalziel et al., 2016). The route of administration was *via* a subcutaneous 2 mL capacity slow-release osmotic mini pump (Durect Corporation, Alzet Osmotic Pumps, Cupertino, CA, USA) as previously described (Dalziel et al., 2016). The control group received DMSO vehicle only *via* the same delivery method. The control group consisted of 13 rats and the loperamide-treated group had 11. Some rats died before the end of the study from age related issues (Dalziel et al., 2017).

### 2.3 Caecal microbiota

Caecal content samples were collected rapidly after euthanasia, using carbon dioxide inhalation overdose, and snap frozen in liquid nitrogen and stored at -80°C before use. Metagenomic DNA was extracted using the NucleoSpin Soil kit (Macherey-Nagel GmbH, Düren, Germany) according to the manufacturer’s instructions, using SL2 lysis buffer and SC enhancer, with the addition of bead beating for four minutes using a BioSpec Mini Beadbeater 96 (Bartlesville, OK, USA) set to maximum speed.

DNA samples were then analysed by 16S rRNA gene amplicon sequencing using the Illumina MiSeq platform with 2 × 250 bp paired-end sequencing with PCR primers targeting the V3 and V4 region (Illumina, 2013):

Forward Primer: 5’-TCGTCGGCAGCGTCAGATGTGTATAAGAGACAGCCTACGGGNGGCWGCAG

Reverse Primer: 5’-GTCTCGTGGGCTCGGAGATGTGTATAAGAGACAGGACTACHVGGGTATCTAATCC

PCR thermal cycler conditions were used as specified in the Illumina library preparation protocol (95°C for 3 minutes; 25 cycles of [95°C for 30 seconds, 55°C for 30 seconds, 72°C for 30 seconds]; 72°C for 5 minutes; Hold at 4°C) (Illumina, 2013). Sequence reads were quality trimmed using the following parameters in QIIME 2 (Bolyen et al., 2019): Adapter sequences were removed using the cutadapt function, paired reads joined using vsearch with a minimum overlap of 20 bp, reads were quality trimmed with a 25 q-score cut off, remaining reads denoised and chimera checked using the deblur algorithm. Single nucleotide variants were classified by aligning against the Silva 132 small subunit ribosomal RNA database. Alpha diversity was assessed using the Faith’s Phylogenetic Diversity and Chao1 index. Beta diversity was compared using Principal Coordinate Analysis (PCoA) of weighted unifrac phylogenetic distances. The sampling depth used for alpha and beta diversity analysis was 32000 reads. Differences in taxa were analysed using ANCOM-BC (Lin and Peddada, 2020) with q<0.05 considered significant. Differences in overall community profiles were analysed by permutation multivariate analysis of variation (PERMANOVA) using the anosim function in the ‘vegan’ package for R. R version 1.4.1103 was used for all statistical analyses (R Core Team, 2021). Data is presented as mean percentage +/- SEM.

Sequence reads can be downloaded from the NCBI Sequence Read Archive (SRA) under accession PRJNA819534.

## 3 Results

### 3.1 Taxonomic composition at the phylum level

**Figure 1:**
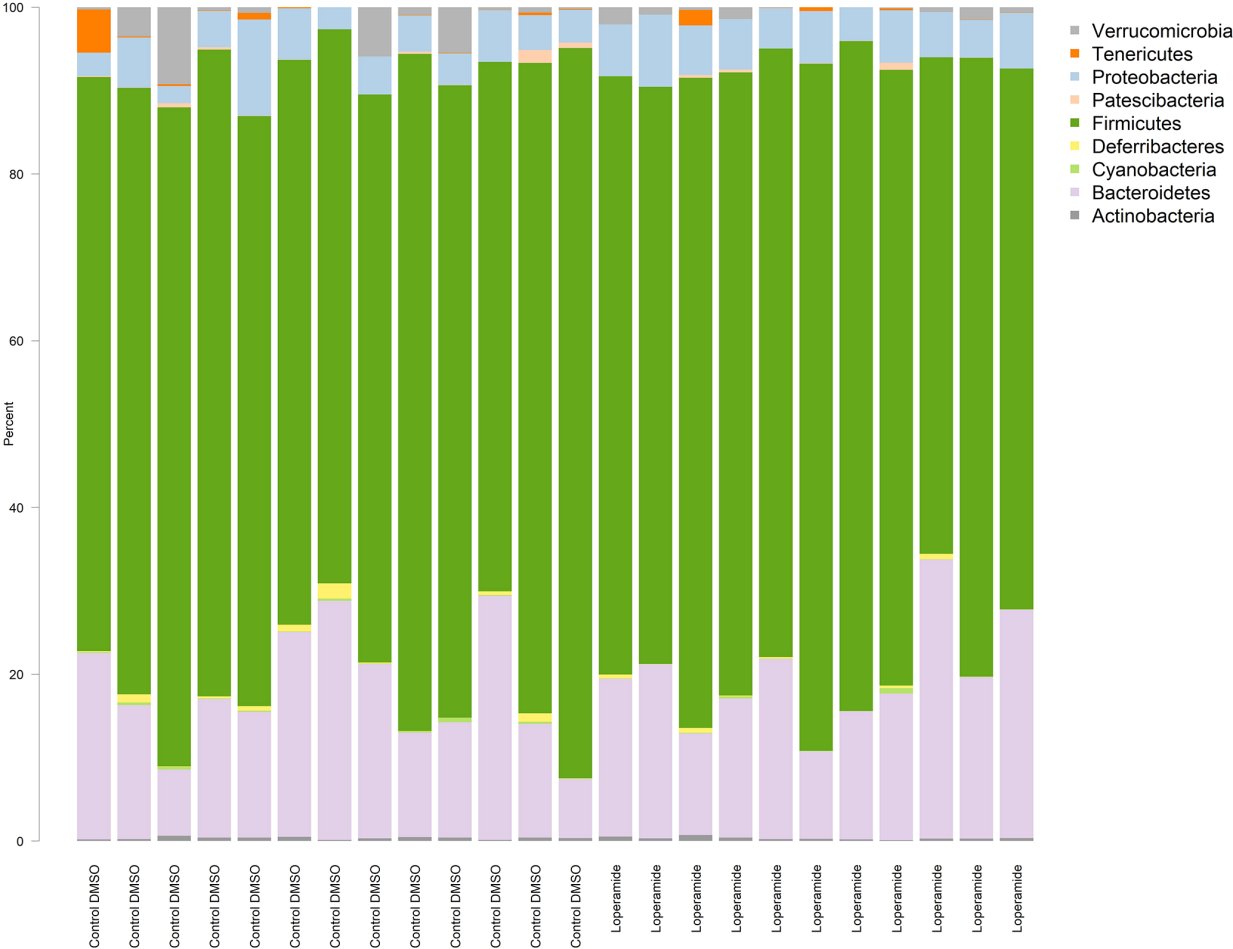
Distribution of the gut microbiota at the Phylum level.

A total of 10 bacterial phyla were detected in both loperamide-treated and control groups, which included four frequently detected phyla: *Bacteroidetes, Actinobacteria, Firmicutes* and *Proteobacteria*, and six minor phyla *Tenericutes, Deferribacteres, Patescibacteria, Cyanobacteria, Elusimicrobia* and *Verrucomicrobia* (**Figure 1**). All 24 samples contained the four frequently found phyla. No more than six samples contained each of the six minor phyla. No clear differences between treatment were observed at the phylum level (q>0.05).

### 3.2 Microbiota at the family and genus levels

Analysis of the microbiota at the family level showed significant differences (q<0.05) between the loperamide-treated and control groups in eleven families (**Table 1**). Of these, approximately half were from the Firmicutes phylum; *Aerococcaceae*, *Carnobacteriaceae*, *Enterococcaceae*, *Streptococcaeae*, *Defluviitaleaceae* and *Lachnospiraceae*, which were the most abundant of the significantly different families (loperamide 25.746% ± 1.94; control 31.69% ± 2.141).

**Table 1:** Families with significantly different relative abundances between loperamide- treated and control rats. Data represented as mean percent ± standard error of mean (SEM).

Phylum	Family	Control	Loperamide	q-value
Bacteroidetes	Marinifilaceae	0.127 ± 0.022	0.337 ± 0.041	0.0009
Proteobacteria	Unclassified Rhodospirillales	0.008 ± 0.003	0.042 ± 0.012	0.03
Proteobacteria	Pseudomonadaceae	0.013 ± 0.002	0.008 ± 0.002	0.01
Actinobacteria	Corynebacteriaceae	0.010 ± 0.003	0.003 ± 0.001	0.03
Cyanobacteria	Unclassified Gastranaerophilales	0.187 ± 0.040	0.111 ± 0.056	0.02
Firmicutes	Aerococcaceae	0.025 ± 0.005	0.010 ± 0.003	0.005
Firmicutes	Carnobacteriaceae	0.187 ± 0.018	0.125 ± 0.017	0.001
Firmicutes	Enterococcaceae	0.066 ± 0.011	0.036 ± 0.005	0.005
Firmicutes	Streptococcaceae	0.329 ± 0.043	0.217 ± 0.030	0.01
Firmicutes	Defluviitaleaceae	0.043 ± 0.007	0.022 ± 0.004	0.01
Firmicutes	Lachnospiraceae	31.69 ± 2.141	25.746 ± 1.94	0.02

Extensive differences at the genus level were also observed with 29 genera significantly different between treatments (**Table 2**). Of the most abundant taxa, *Roseburia* (loperamide 1.527% ± 0.695; control 4.489% ± 0.844) and Unclassified *Lachnospiraceae* (loperamide 2.655% ± 0.649; control 7.575% ± 1.168) were significantly lower in loperamide-treated rats. Genera that were significantly more abundant in the loperamide-treated group and that had a mean relative abundance greater than 1% included *Bacteroides*, *Phascolarctobacterium*, *Ruminococcaceae* UCG-005, *Lactobacillus*, *Blautia*, *Christensenellaceae* R-7 group and the *Ruminococcaceae* NK4A214 group.

**Table 2:** Genera with significantly different relative abundances between loperamide- treated and control rats. Data represented as mean percent ± standard error of mean (SEM).

Family	Genus	Control	Loperamide	q-value
Bifidobacteriaceae	Bifidobacterium	0.007 ± 0.002	0.041 ± 0.017	0.036
Bacteroidaceae	Bacteroides	4.964 ± 0.709	7.731 ± 1.113	0.013
Ruminococcaceae	Candidatus Soleaferrea	0.029 ± 0.006	0.037 ± 0.005	0.014
Barnesiellaceae	Barnesiella	0.027 ± 0.010	0.052 ± 0.012	0.013
Ruminococcaceae	Ruminococcaceae NK4A214 group	0.912 ± 0.108	1.427 ± 0.163	<0.001
Ruminococcaceae	Ruminococcaceae UCG-005	3.789 ± 0.751	7.616 ± 1.177	0.003
Ruminococcaceae	Ruminococcaceae UCG-010	0.221 ± 0.036	0.072 ± 0.016	0.003
Marinifilaceae	Butyricimonas	0.096 ± 0.016	0.235 ± 0.033	<0.001
Marinifilaceae	Odoribacter	0.031 ± 0.007	0.102 ± 0.015	<0.001
Erysipelotrichaceae	Erysipelotrichaceae UCG-003	0.220 ± 0.072	0.623 ± 0.135	0.013
Erysipelotrichaceae	Faecalibaculum	0.068 ± 0.045	0.155 ± 0.040	0.023
Erysipelotrichaceae	Unclassified Erysipelotrichaceae	0.536 ± 0.159	0.054 ± 0.041	0.007
Acidaminococcaceae	Phascolarctobacterium	4.701 ± 1.213	8.507 ± 1.172	0.025
Unclassified Rhodospirillales	Unclassified Rhodospirillales	0.008 ± 0.003	0.042 ± 0.012	0.003
Burkholderiaceae	Parasutterella	0.327 ± 0.074	0.535 ± 0.069	0.003
Enterobacteriaceae	Kluyvera	0.001 ± 0.0003	0.002 ± 0.001	0.015
Carnobacteriaceae	Granulicatella	0.002 ± 0.001	0	0
Lactobacillaceae	Lactobacillus	3.469 ± 0.642	7.088 ± 0.809	0.001
Christensenellaceae	Christensenellaceae R-7 group	0.940 ± 0.096	1.688 ± 0.259	0.003
Atopobiaceae	Unclassified Atopobiaceae	0.004 ± 0.001	0.012 ± 0.003	0.014
Family XIII	Anaerovorax	0.047 ± 0.010	0.056 ± 0.004	0.021
Lachnospiraceae	ASF356	0.180 ± 0.1	0	0
Lachnospiraceae	Blautia	3.251 ± 1.132	5.246 ± 0.874	0.025
Eggerthellaceae	Enterorhabdus	0.001 ± 0.001	0	0
Lachnospiraceae	GCA-900066575	0.259 ± 0.049	0.083 ± 0.028	0.042
Eggerthellaceae	Gordonibacter	0.009 ± 0.001	0.013 ± 0.002	0.013
Lachnospiraceae	Marvinbryantia	0.243 ± 0.069	0.912 ± 0.213	<0.001
Lachnospiraceae	Roseburia	4.489 ± 0.844	1.527 ± 0.695	0.013
Lachnospiraceae	Unclassified Lachnospiraceae	7.575 ± 1.168	2.655 ± 0.649	0.014

### 3.3 Microbiota diversity

**Figure 2:**
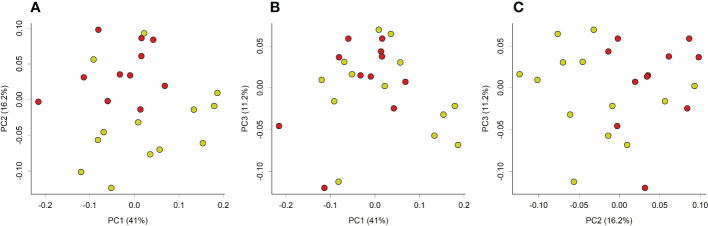
Principal coordinate analysis (PCoA) plot of weighted unifrac phylogenetic distances of caecal microbiotas from control (yellow) or loperamide (red) groups. Plots show **(A)** PC1 vs PC2, **(B)** PC1 vs PC3, and **(C)** PC2 vs PC3. Percentages on axes indicate proportion of variation explained by each dimension. Communities between groups were significantly different (PERMANOVA P=0.008).

**Figure 3:**
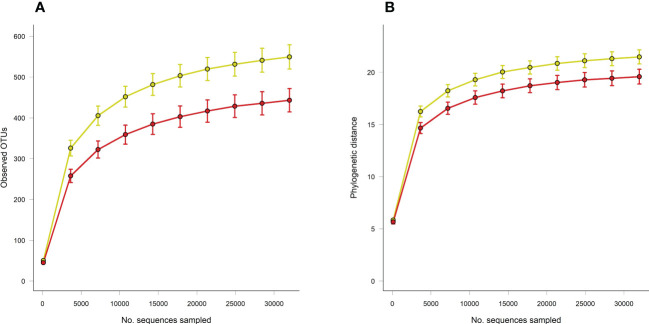
Rarefaction curves of **(A)** observed OTUs and **(B)** Faith’s phylogenetic distance (PD) between caecal microbiotas from control (yellow) or loperamide-treated (red) groups. Error bars show SEM of 10 iterations per sampling depth. Observed OTUs P=0.017, Faith’s PD P=0.06.

Principal coordinate analysis of Unifrac phylogenetic distances showed strong separation overall between caecal microbiotas from control and loperamide-treated rats (**Figure 2**). Permutation multivariate analysis of variance (PERMANOVA) confirmed that the overall differences in communities were significant (P=0.008). The microbiotas of control rats were also significantly more diverse with more observed operational taxonomic units (OTUs) (P=0.017), and a higher Faith’s phylogenetic distance (P=0.06) compared to loperamide-treated rats (**Figure 3**).

## 4 Discussion

It is becoming increasingly important to understand the association between GI transit time and the gut microbiota, due to the potential impact of the gut microbiota on host physiology and the transition between healthy and diseased states. We previously reported that the pharmacological drug loperamide delayed GI transit in rats compared to un-treated (Dalziel et al., 2017). Delayed GI transit time, as seen in people with constipation, affects the microbiota composition by decreasing beneficial bacteria and increasing harmful bacteria (Zhao and Yu, 2016). Interestingly, *Bacteroides*, the predominant genus in the human gut and a beneficial symbiont/commensal (Wexler, 2007), in this study was found to be more relatively abundant in loperamide-treated rat caecal samples compared to controls. This finding is consistent with a previous study in which *Bacteroides* were shown to be significantly increased in constipated women compared to controls (Li et al., 2021). The loperamide-induced prolonged transit time (Dalziel et al., 2017) might have facilitated the increase in relative abundance of *Bacteroides*, indicating that they can adapt well in a slow and competitive environment (Based on r/K selection theory of microbial ecology) (Pianka, 1970). Increased relative abundance of *Bacteroides* may be associated with alteration of gut microbiota homeostasis. Given their large genome bank, *Bacteroides* have the ability to turn on certain genes to shift from friendly commensal to harmful bacteria (Wexler, 2007).

In contrast, loperamide-induced slowed transit caused a decrease in the relative abundance of the phylum *Actinobacteria* and selected genus in the phylum *Firmicutes* (*Roseburia*) that are associated with faster colonic transit (Parthasarathy et al., 2016). These findings suggest that taxa of these phyla do not adapt well in a slowed transit luminal environment. Overall, these findings suggest that normal gut motility is key in maintaining a balanced gut ecosystem and gut homeostasis.

In this study, we investigated if changes in microbiota is associated with changes in gut transit time (using data from a previous study where loperamide was effective at inhibiting GI transit compared to controls) (Dalziel et al., 2017). We found that the ecological diversity and richness in the caecal microbiota differed significantly between loperamide-treated rats and controls. Alpha-diversity analysis showed that the richness and diversity of the bacterial communities was significantly lower in the loperamide-induced slowed transit group compared to the control group. This is in line with a study by Ren et al., who showed that the control group exhibited higher bacterial diversity and richness than the constipation group, concluding that higher microbial diversity may correspond to healthier ecosystems (Ren et al., 2017). In contrast, several studies have reported the diversity and richness of bacterial communities to be higher in the constipation group compared to controls (Li et al., 2020; Müller et al., 2020). Furthermore, these studies went on to show that increased alpha diversity was significantly associated with longer colonic passage, the explanation being diversification as an adaption to a perturbed ecosystem (i.e., depletion of nutrients, switch from microbial saccharolytic to proteolytic fermentation, microbial competition and decreased water availability) (Müller et al., 2020). These contrasting findings indicate that microbial diversity should be interpreted within the physiological context and reduced microbial alpha diversity should not necessarily be represented as reduced microbiota stability.

Numerous studies have documented the role of gut microbiota-derived molecules in regulating gut motility (Dalile et al., 2019; Sun et al., 2019). Production of short chain fatty acids (SCFA), especially butyrate, by the gut microbiome was shown to influence GI motility (Cherbut et al., 1997). In our study, the butyrate producing genus *Roseburia* was found to be significantly reduced in loperamide-induced constipated rats. This is similar to a study by Chassard et al. in which butyrate-producing *Roseburia - E. rectale* group was found to be lower in the IBS-with-constipation group compared to controls. The authors concluded that a reduced relative abundance of butyrate producers makes colonic transit slower (Chassard et al., 2012). Experiments by Soret et al., and Reigstad et al., showed that butyrate producing bacteria may increase colonic motility by inducing the release of serotonin or promoting cholinergic pathways (Soret et al., 2010; Reigstad et al., 2014). Conversely, studies have reported butyrate producing genera to be associated with constipation (Yarullina et al., 2020). Butyrate has been shown to impact various colonic effects; such as inhibition of smooth muscle contractions in the colon, reduction of stool volume through stimulation of colonic electrolyte and water absorption, predisposing to constipation (Yarullina et al., 2020). These inconsistencies in the literature can be addressed by carrying out further research to identify the mechanisms and involvement of butyrate producers in prolonged colonic transit.

We speculate that a slowed gut transit might modify the spatial organization and proportion of the microbiota by creating a luminal microenvironment for the growth of specific bacterial taxa, or by affecting bacterial colonization. Moreover, the influence of luminal microenvironments might be relevant in regions where key motility patterns are initiated such as the proximal colon. In our study, a slowed GI transit time induced by loperamide led to increased relative abundance of families *Bacteroidaceae* and *Marinifilaceae*, belonging to the phylum Bacteroidetes. Several studies have proposed Bacteroidetes to be the dominant gram-negative bacteria in the GI tract (Eckburg et al., 2005). Alterations in distribution of gram-negative bacteria is associated with elevated levels of lipopolysaccharides (LPS), a cell wall component of gram-negative bacteria (Salguero et al., 2019). LPS is thought to be an important mediator of the microbiome’s influence on host physiology. Several studies have pointed out an inhibitory role of LPS on GI motility. Mikawa et al., through their experiments showed that LPS-induced nitric oxide synthase produced nitric oxide, which in turn inhibited GI motility (Mikawa et al., 2015). It would therefore follow that changes in the composition of gram-negative bacteria might further cause GI motility disturbances. We did not measure LPS levels in this study; future studies might shed more light on the possible specific association of gram-negative bacteria and LPS on GI motility.

In the present study, we used 16S rRNA gene amplicon sequencing to analyse DNA samples. Although there is valuable information gained from 16S sequencing, there are also some limitations. The sequencing depth may not be sufficient for short amplicon sequencing to capture novel or low abundance microbial species. Moreover, this method does not directly provide information about the functional capacities of the organisms. In contrast, whole genome sequencing would have revealed a strain level resolution of both microbiota abundance and functional capacity and would have given a comprehensive understanding regarding the association between varied gut transit time and dysbiosis. This could be the subject of further studies.

## 5 Conclusion

Our findings indicate that the loperamide-induced alterations in gut transit time affected the diversity and relative abundance of caecal microbial communities. We speculate that slowed colonic transit facilitates the amplification and colonization of select genera such as *Bacteroides* that adapt well in slow and competitive environments (corresponding to prolonged transit and limited resources). The relationship between gut motility and microbiota is relevant in experimental models used to study several functional GI disorders associated with the gut microbial composition, such as IBS and constipation, where GI transit is also altered. Understanding the link between specific microbial species and varying transit times is crucial to design microbiota-based interventions to treat intestinal motility disorders.

## Data availability statement

The datasets presented in this study can be found in online repositories. The names of the repository/repositories and accession number(s) can be found below: https://www.ncbi.nlm.nih.gov/, PRJNA819534.

## Ethics statement

The animal study was reviewed and approved by AgResearch Grasslands Animal Ethics Committee.

## Author contributions

NP, JD, and WY contributed to conception and design of the study. WY organized the database and performed the statistical analysis. NP wrote the first draft of the manuscript. All authors contributed to the article and approved the submitted version.
